# *Escherichia coli*-related disseminated intravascular coagulation: Case report and literature review

**DOI:** 10.1097/MD.0000000000032750

**Published:** 2023-02-17

**Authors:** Junjun Wu, Huaming Li, Yufang Wang, Rong Xu

**Affiliations:** a Department of Gastroenterology, Hangzhou Third People’s Hospital, Hangzhou, Zhejiang, China.

**Keywords:** coagulation abnormalities, coagulation disorders, case report, DIC, *Escherichia coli*, procalcitonin, sepsis, thrombocytopenia

## Abstract

*Escherichia coli* can cause severe infections. The latter can lead to disseminated intravascular coagulation (DIC). The importance of an early diagnosis of DIC is illustrated through this case report.

**Aim:**

Review the utility and shortcomings of representative clinical indicators of *E coli* infection and DIC.

**Case report:**

A 48-year-old man presented with diarrhea, nausea, and vomiting with fever of 2-day duration, during which consciousness was lost for 12 hour. Hematology was undertaken. The coagulation profile, liver function, and kidney function were determined, and blood cultures undertaken. The final diagnosis was acute gastroenteritis complicated by DIC. Meropenem (1.0 g, q8h, i.v.) was started, along with active replacement of fluids. Anticoagulant therapy (low-molecular-weight heparin 0.4 mL, q.d.s.) was given. Plasma supplementation of coagulation factors and albumin was applied. On day-5 of therapy, hematology showed the platelet count, D-dimer level, and prothrombin time to be improved significantly. Low-molecular-weight heparin treatment was stopped and antibiotic treatment was continued for 1 week. The patient made a full recovery.

**Conclusions:**

In severe infection, timely assessment of the platelet count, procalcitonin level, coagulation function, as well as rational use of antibiotics, can improve the prognosis of patients.

## 1. Introduction

Sepsis is a life-threatening condition that arises when the body’s response to infection causes injury to its own tissues and organs. Sepsis can also cause multiple-organ dysfunction syndrome. Sepsis can lead to admission to the intensive care unit, and mortality from sepsis can kill 30% to 50% of patients suffering from it.^[1,2]^

In the early stages of sepsis, a clotting cascade triggered by activation of tissue factors initiates the exogenous clotting pathway.^[3]^ Prothrombin and thrombin are activated by tissue factors compounded with factor VII. The resulting impairment of anticoagulation and fibrinolysis can lead to microvascular thrombosis and consume various coagulation factors. These actions lead to disseminated intravascular coagulation (DIC), which further promotes ischemia and hypoxia, tissue damage, and multiple-organ failure.

Early and timely diagnosis of organ injury can delay the occurrence and development of DIC. Combination of clinical data with laboratory tests and scoring according to International Society on Thrombosis and Hemostasis guidelines can improve the prognosis of patients with DIC.

Here, we report the data of a 48-year-old man with diarrhea, nausea, vomiting, fever, and multiple-organ insufficiency due to *Escherichia coli* infection. We review the utility and shortcomings of representative clinical indicators of *E coli* infection and DIC.

## 2. Case report

The patient was a 48-year-old man, presented with diarrhea with fever of 2-day duration. During this time, he lost consciousness for 12 hour. Two hours after eating cured meat, the patient had watery stools, nausea, chills and fever, and vomited his stomach contents; the highest body temperature reached was 38.3°C He self-administered Tylenol™ tablets. Symptoms were relieved, and he did not suffer chest tightness, cough/phlegm, headache, dizziness, hematemesis, black stools, abdominal pain, or other discomfort.

The next morning, 2 hour after eating cured meat, the symptoms stated above recurred. Convulsions, foaming at the mouth, and other discomfort were not observed. His family members sent him to the emergency department of our hospital.

Obvious abnormalities were not found upon computed tomography of the head. He was treated with cefoperazone sodium and sulbactam sodium (intravenous drip) to counteract an infection. Dexamethasone was given to ablate inflammation. Fluids were administered to aid rehydration. He was admitted to our department for further treatment.

### 2.1. Medical history and family history

He had been taking amlodipine besylate (5 mg, p.o.) once-daily for long-term treatment of hypotension. Good control of blood pressure had been documented. He nor his family had a history of drug allergy.

### 2.2. Physical examination upon hospital admission

The patient had a body temperature of 37.4°C, respiratory rate of 20 times/minute, blood pressure of 116/76 mm Hg, and pulse of 84 times/minute. Skin temperature was normal, purple lines and “flower spots” were absent, and normal circulation of extremities was documented. Thick breathing sounds in both lungs and a wheezing sound at the end of exhalation in the left lung were noted, but no obvious dry or wet rales in either lung. The heart rate was 84 times/min with a heart murmur was absent. Tenderness and rebound pain in the whole abdomen were not observed, and bowel sounds were 6 times/minute. Edema in the lower extremities was not observed.

### 2.3. Laboratory tests

In the emergency department, hematology revealed a white blood cell count of 12.9 × 10^9^/L, hemoglobin level of 165 g/L, platelet count of 202 × 10^9^/L, percent neutrophils of 76.4%, and C-reactive protein (CRP) level of 105.3 mg/L. Levels of procalcitonin of 37.454 ng/mL, alanine aminotransferase of 273 U/L, aspartate aminotransferase of 520 U/L, lactate dehydrogenase of 756 U/L, creatinine of 153 μmol/L, and troponin of 1.921 ng/mL were documented. In terms of coagulation function, the prothrombin time (PT) was 12.3 seconds, International Normalized Ratio (INR) was 1.1, and D-dimer level was 6.56 mg/L. Electrocardiography indicated a sinus rhythm.

Upon admission to hospital, antibiotic (meropenem, 1.0 g, q8h, i.v.) treatment was initiated. Three days after treatment initiation, hematology revealed the CRP level and percent neutrophils (89.8%) to be increased (108.0 mg/L), and white blood cell count (8.8 × 10^9^/L), red blood cell count (4.18 × 10^12^/L), hemoglobin level (123 g/L), and platelet count (33 × 10^9^/L) to be decreased. Levels of alanine aminotransferase (1265 U/L), aspartate aminotransferase (2563 U/L), creatine kinase (4585 U/L), creatine phosphokinase-MB (62 U/L), creatinine (92 μmol/L), glucose (9.28 mmol/L), lactate dehydrogenase (704 U/L), and troponin (1.522 ng/mL) were increased, whereas the potassium level (3.36 mmol/L) was reduced. Levels of direct bilirubin (33.0 μmol/L), glutamyl phthalate (156 U/L), α-hydroxybutyrate dehydrogenase (596 U/L), indirect bilirubin (33.4 μmol/L), total bilirubin (66.4 μmol/L), procalcitonin (26.469 ng/mL), B-type natriuretic peptide precursor (1348.0 pg/mL), D-dimer (15.68 mg/L), fibrinogen (4.02 g/L), as well as PT (14.1 seconds), were increased.

Sepsis with dominant DIC was considered. Four days after treatment initiation, the laboratory report stated that blood culture was positive for *E coli*.

### 2.4. Imaging

Ultrasound of the liver, bile, pancreas, and spleen indicated fatty liver disease. Ultrasound of the urinary system showed no obvious abnormality.

### 2.5. Final diagnosis

The final diagnosis was acute gastroenteritis complicated by DIC.

### 2.6. Treatment

**Figure 1.**
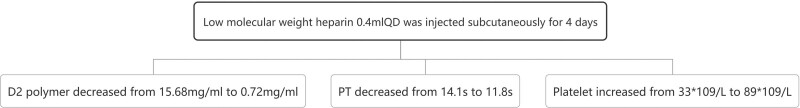
Changes in coagulation function and platelet count after anticoagulant therapy.

**Figure 2.**
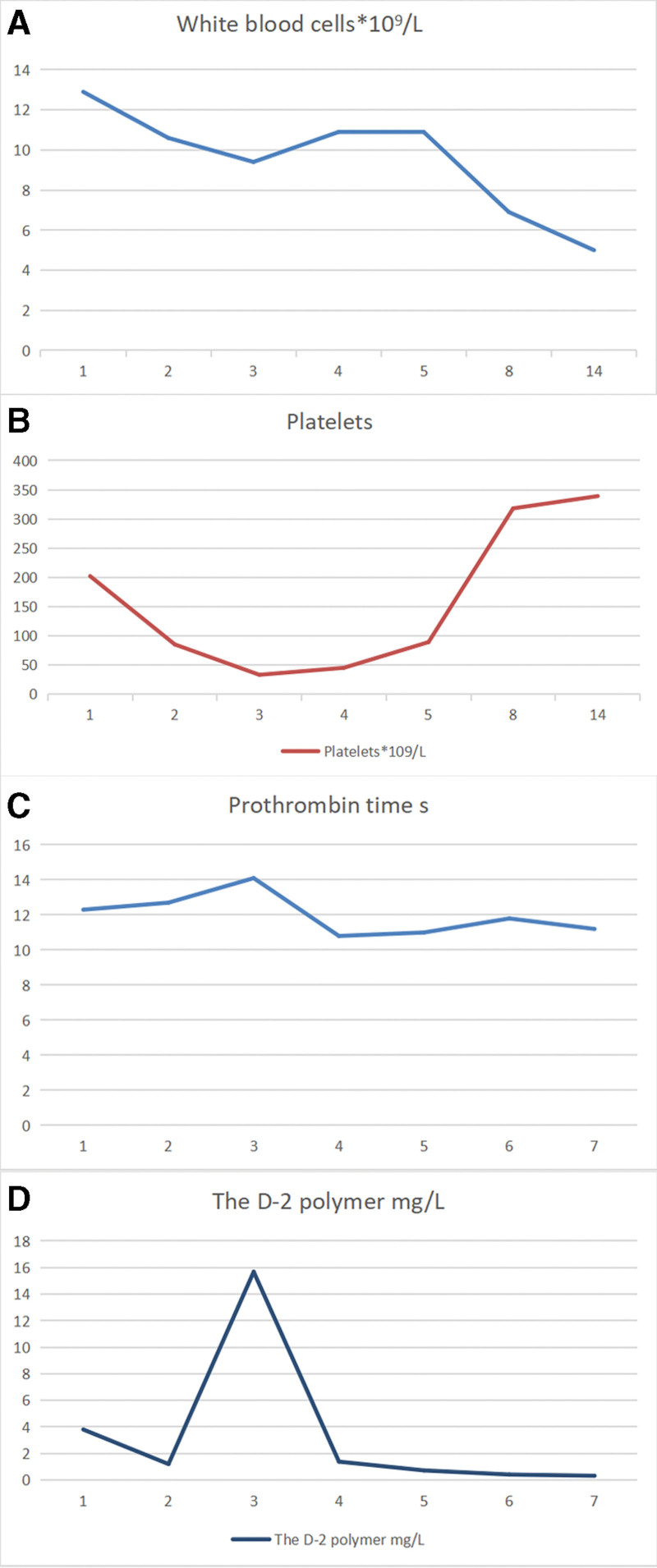
Changes in the leukocyte count, platelet count, prothrombin time, and D-dimer level after anticoagulation treatment (low-molecular-weight heparin).

After hospital admission, meropenem (1.0 g, q8h, i.v.) was started, along with active replacement of fluids. DIC was diagnosed on day-3 of treatment. Anticoagulant therapy (low-molecular-weight heparin, 0.4 mL, q.d.s.) was given (Fig. 1). Meanwhile, plasma supplementation of coagulation factors and albumin was applied. On day-5 of therapy, hematology showed the platelet count, D-dimer level, and PT to be improved significantly (Fig. 2A–D). Treatment with low-molecular-weight heparin was stopped and antibiotic treatment was continued for 1 week. Repeat hematology showed the CRP level and procalcitonin to be improved (Fig. 2A–D).

Table 1 describes the laboratory results from day 1 to day 14 after the event.

**Table 1:** Describes the laboratory results from day 1 to day 14 after the event.

Blood laboratory Blood data	Day1	Day2	Day3	Day4	Day5	Day8	Day14 discharge	Day 22 Follow up
WBC 10^9^/L (3.5–9.5)	12.9	10.6	8.8	10.9	10.2	6.9	5	
PLT 10^9^/L (125–350)	202	85	33	45	89	318	335	179
HB g/L (130–175)	165	120	123	128	129	123	119	150
CRP mg/L (0–10)	105.3	99	108	56.3	3.9	<0.5	<0.5	7.3
N% (40–75)	76.4	94.8	89.8	85	53.7	48.4	48.5	81
Procalcitonin ng/mL (0–0.046)	37.454	39.876	47.471	26.469	1.722	0.073	0.029	
ALT U/L (0–50)	273	542	1265	871	365	114	42	<0.01
AST U/L (15–40)	520	2563	483	409	80	31	22	28
Tot. Bilirubin μmol/L (0–26.0)		39	66.4	37.5	18.4	20.8	13	
Direct Bilirubin μmol/L (0–8.0)		20.3	33.4	15.6	8.3	7.5	4.6	
Potassium mmol/L (3.50–5.30)	3.47	3.19	3.36	3.92	3.43	4.24	3.64	
GGT U/L (10–60)		103	156	139	144	89	51	
Creatinine μmol/L (57–97)	153	82	117	78	81	91	94	
LDH μmol/L (120–250)	296	2491	1462	404	239	193	127	168
Troponin ng/mL (0.0–0.040)	0.088	1.921	1.857	0.409	0.156	0.015		
Glucose mmol/L (3.90–6.10)	9.37	8.6	9.28	9.33	7.22	4.98	4.22	6.34
Fibrinogen g/L (2.0–4.0)	3.53	3.34	4.02	5.13	4.9	4.02	3.49	
APTT s (22.0–35.0)	27.1	31.6	35.8	31.3	28.3	28.6	28.1	
PT s (9.0–14.0)	12.3	12.7	14.1	10.8	11	11.8	11.2	
INR (0.8–1.24)	1.1	1.14	1.26	0.96	0.98	1.05	1	
D-Dimer mg/L (0.00–0.55)	6.56	1.22	15.68	1.38	0.72	0.41	0.32	

ALT = alanine aminotransferase, AST = aspartate aminotransferase, CRP = c-reactive protein, INR = international normalized ratio, LDH = lactate dehydrogenase, WBC = white blood cell.

### 2.7. Outcome and follow-up

The patient was cured and discharged 22 days after hospital admission. At 1-month follow-up, hematology findings, coagulation function, the biochemical profile, and myocardial-enzyme profiles were within the normal range.

## 3. Discussion

Our middle-aged patient developed the gastroenteritis-related symptoms of nausea, vomiting, and diarrhea due eating undercooked cured meat, did not pay attention to the early signs of dehydration, and progressed to a shocked state. Upon hospital admission, he presented with hypovolemic shock. Laboratory tests suggested multiple-organ dysfunction. After hospital admission, hematology suggested progressive decline in the platelet count, increase in D-dimer level, PT prolongation, and hypofibrinogenemia. Subsequent blood-culture results suggested sepsis-dominant DIC. Distinguishing sepsis from DIC is very important for the early diagnosis, early treatment, and improved prognosis of DIC patients.

If thrombocytopenia occurs, megakaryocytes produce platelets to maintain the physiological hemostasis of the body. If body tissue is damaged, substances are released to shrink blood vessels. Platelets also activate the coagulation system so that the fibrinogen in plasma is converted to fibrin and, finally, the fibrin network is formed. Thrombocytopenia also mediates inflammation,^[4,5]^ and the immune response. The causes of thrombocytopenia include decreased production,^[6]^ increased consumption, or increased destruction of platelets in the spleen. Thrombocytopenia in severe infection is an independent risk factor for severe disease. Platelets can induce neutrophils to capture pathogenic microorganisms.^[7]^ Also, platelet–neutrophil interactions induce transcellular synthesis and overactivation of neutrophils, resulting in increased secretion of proinflammatory molecules. In mice, researchers^[8]^ have shown that platelet loss promotes the spread of bacteria, leading to increased inflammation throughout the body and an increased risk of death. Among them, the toll-like receptor 2 (rs111200466) and toll-like receptor 4 (rs11536889) variants,^[9]^ in platelets are prognostic factors for DIC, which can lead to severe organ dysfunction and high mortality. Therefore, platelets play an important part in severe infection.

### 3.1. Clotting abnormality

Sepsis-associated DIC is characterized by reduced levels of endogenous coagulation inhibitors (including antithrombin). However, the KyberSept tria^[10]^ failed to demonstrate that use of high doses of antithrombin had a beneficial effect upon mortality in patients with severe sepsis. Tagami and colleagues^[11]^ reintegrated national data and found that a supplemental dose of antithrombin was efficacious against sepsis-related DIC.

Cytokines mediate coagulation disorders in systemic inflammatory activities. Tissue factors are the main moieties that mediate thrombin generation and the imbalance/dysfunction of normal physiological anticoagulation mechanisms.^[12]^ Coagulation function can also affect inflammatory activities.^[13,14]^ The most important manner in which thrombin affects inflammation is binding of proteinase-activated receptors (PARs). Four types of PAR (1–4) have been identified, and all belong to a family with transmembrane domains: G-protein-coupled receptors.^[15]^ Activating PARs induces a pro-inflammatory response.^[16,17]^

### 3.2. Disseminated intravascular coagulation

DIC is not an isolated disease. It is often secondary to other diseases, such as severe infection, tumor, major obstetric events, severe trauma, immune reaction, heat shock, etc.^[18]^ In 2001, the International Society for Thrombosis and Hemostasis defined DIC as “an acquired syndrome characterized by activation of intravascular coagulation and loss of location due to various causes that may originate from and lead to damage to the microvascular system and, if severe enough, organ dysfunction.^[10]^ The diagnostic criteria for DIC are proposed in this guideline. Refer to the following table. In the simplified version of DIC diagnosis, although there is no difference in mortality between the 2 groups, sepsis-related coagulation disorders may be valuable in detecting suitable candidates for anticoagulant therapy in sepsis.^[18]^ DIC is significantly higher in judging disease severity and mortality than non-DIC patients, but it cannot predict the results after adjusting for disease severity.^[19]^

In addition to pathogen-induced coagulation activation, other important pathways (e.g., injury-related molecular patterns, neutrophil extracellular traps, extracellular vesicles,^[20]^ glycocalyx injury) are also involved in the pathogenesis of sepsis-induced DIC.^[21]^ In addition, experiments have revealed that obese piglets showed severe DIC and pro-coagulant reactions, and that obesity aggravated the occurrence and development of DIC.^[3]^

### 3.3. Procalcitonin

Procalcitonin is an intracellular precursor of calcitonin, secreted by thyroid C cells, and can be used as a biomarker of bacterial, viral, and non-bacterial infections. Under normal circumstances, procalcitonin > 0.25 ng/mL can indicate bacterial infection. Studies have shown that although the procalcitonin level cannot guide when antibiotic therapy should be started, it is valuable for knowing when to discontinue antibiotic therapy.^[22]^ Guidelines for sepsis management set by Sager R et al in 2016 stated that the procalcitonin level could be used as a reference index to shorten the course of antibiotic therapy, but that it should be combined with clinical data.^[23]^

The CRP level is increased in viral and noninfectious diseases. The specificity and accuracy of procalcitonin and CRP in sepsis have been compared.^[24]^

An increase in the procalcitonin level should be combined with data on the renal function and cardiac function of patients.^[25]^ Even though procalcitonin is cleared not only in the kidneys, in chronic kidney disease, the procalcitonin level can be increased even if infection is absent.^[26]^ Even though procalcitonin is cleared not only in the kidneys, in chronic kidney disease, the procalcitonin level can be increased even if infection is absent.^[27]^ Even though procalcitonin is cleared not only in the kidneys, in chronic kidney disease, the procalcitonin level can be increased even if infection is absent.^[27]^

### 3.4. Escherichia coli

*E coli* is a common pathogenic bacterium found in the bloodstream infections of hospitalized patients.^[28]^ It infects surgical sites, the urinary tract, and pelvis.^[29]^ Thomas-Rüddel and colleagues showed that in patients with peritoneal dialysis-associated peritonitis, patients with *E coli* infection were more likely to need mechanical ventilation and to be hospitalized.^[30]^ Also, patients with extended-spectrum beta-lactamase-producing *E coli* tend to have longer hospitalization due to catheter-associated infection.^[30]^

Antibiotics are used widely, which has led to the problem of antibiotic resistance. For an infection caused by *E coli*, the selection of antibiotics is also a difficult problem. In vitro experiments have suggested that carbapenem antibiotics are efficacious against *E coli*, but the sensitivity of *E coli* is decreasing.^[32]^ In carbapenem-resistant *E coli*, the resistance of *E coli* is also increasing gradually.^[33,34]^ Selection of efficacious antibiotics, whether to use a combination of drugs or a single drug, and how to monitor the effects of antibiotics are important issues.

## 4. Conclusions

This case study has shown that, in severe infection, timely assessment of the platelet count, procalcitonin level, coagulation function, as well as rational use of antibiotics, can improve the prognosis of patients.

## Acknowledgments

The authors appreciate the patient’s consent to present this case.

## Author contributions

**Conceptualization:** Junjun Wu.

**Formal analysis:** Junjun Wu.

**Investigation:** Rong Xu.

**Methodology:** Junjun Wu.

**Project administration:** Huaming Li, Yufang Wang.

**Visualization:** Yufang Wang.

**Writing – original draft:** Junjun Wu.

**Writing – review & editing:** Yufang Wang.
